# Assistive Technology Needs and Measurement of the Psychosocial Impact of Assistive Technologies for Independent Living of Older Hispanics: Lessons Learned

**DOI:** 10.3390/technologies4030021

**Published:** 2016-07-21

**Authors:** Elsa M. Orellano-Colón, Jeffrey Jutai, Angélica Santiago, Víctor Torres, Keyla Benítez, Mayra Torres

**Affiliations:** 1 Occupational Therapy Program, School of Health Professions, Medical Sciences Campus, University of Puerto Rico, San Juan, PR 00926-1117, Puerto Rico; 2 Puerto Rico Assistive Technology Program, Office of the Vice President for Research and Technology, University of Puerto Rico, San Juan, PR 00926-1117, Puerto Rico; 3 Interdisciplinary School of Health Sciences, University of Ottawa, 25 University St., Ottawa, K1N 6N5, Ontario, ON, Canada

**Keywords:** assistive technology measurement, psychosocial impact, Hispanics, older adults

## Abstract

(1) Knowledge about the assistive technology (AT) needs and psychosocial impact of AT in different populations is needed because the adoption, retention, or abandonment of AT may be influenced by the psychosocial impact that AT has on its users. The aims of this study were to: (a) identify the AT needs of a sample of Hispanic older adults with functional limitations, (b) describe the psychosocial impact of these technologies on the sample’s quality of life, and (c) describe the methodological challenges in using the Puerto Rican version of the Psychosocial Impact of Assistive Device Scale (PR-PIADS) with a Hispanic sample. (2) Methods: This study used a cross-sectional design conducted with a sample of 60 participants. Data was collected using the Assistive Technology Card Assessment Questionnaire (ATCAQ) and the PR-PIADS. Data analyses included descriptive statistics and bivariate analysis. (3) Results: The sample’s most frequently reported needs for AT devices were in the areas of cooking, home tasks, and home safety activities. The sample reported a positive impact of AT use in their quality of life. Several methodological challenges of the PIADS were identified. (4) Conclusions: The sample has unmet needs for using AT devices to overcome difficulties in daily living activities.

## 1. Introduction

People are living longer, with chronic conditions [1], which are the leading causes of death, disabilities, and health care costs in the US [2,3]. Older people with chronic conditions often experience diminished quality of life, generally because of a long period of functional decline and disability. This can affect a person’s ability to perform important and essential activities, such as cooking, taking medications, or getting dressed [1]. Loss of the ability to care for oneself means further loss of safety and independence, often leading to institutionalization [4].

Unfortunately, disabilities do not occur uniformly among racial and ethnic groups. Sizable racial and ethnic disparities in late-life disabilities in independent living exist, with much higher rates reported among older Hispanics living in Puerto Rico (29.9%) than among older Hispanics (20.6%) and non-Hispanic whites (15.0%) living in the US for year 2012 [5]. One way to reduce older adults’ difficulties in activities and increase their safety and quality of life is by using assistive technology devices (AT) [6–13]. However, Hispanic populations with disabilities in the US, including Puerto Ricans, have reported a lower usage rate of AT as compared to non-Hispanic whites with disabilities [14–16].

One way to eliminate existing health disparities in the use of AT is to understand the AT needs and the psychosocial impact of these devices on the quality of life (QoL) of older Hispanics with functional limitations. New knowledge about the AT needs and psychosocial impact of AT in different populations is needed because the adoption, retention, or abandonment of AT may be influenced by the psychosocial impact that AT has on its users. Moreover, the assessment of the impact of AT in different populations is significant because people’s reactions to their devices are complex and individual [17]. These reactions vary depending on the individuals’ specific needs, abilities, preferences and previous experiences. For example, people with neurological conditions in previous studies have perceived their AT, mostly wheelchair devices, to have a higher impact on improving their sense of competence, independence and motivation to participate in activities than on improving their self-esteem [18–20]. On the other hand, adults 75 years and older with amyotrophic lateral sclerosis have rated the impact of writing aids higher on their self-esteem than on their sense of independence and motivation to participate in activities [21]. These studies validate the fact that assistive devices hold different meanings for different users and there are several possible reasons for using or not using them [22].

There is a knowledge gap in the AT needs as well as in the impact that AT for everyday life activities has on the quality of life (QoL) of older Hispanics who live independently with functional limitations. Since the psychosocial impact associated with the use of AT is an important aspect that determines its integration into the daily life of the user [23], the evaluation of the effect of these devices on quality of life as an outcome measure is important to optimize the process of prescribing and providing AT [24]. Based on this, the purpose of the present study was to (1) describe the AT needs of a sample of Hispanic older adults with functional limitations, (2) describe the psychosocial impact of AT on the dimensions of adaptability, competence, and self-esteem, as reported by a sample of older adults living in Puerto Rico and measured by the Puerto Rican version of Psychosocial Impact of Assistive Device Scale, and (3) identify methodological challenges and lessons learned in using the Puerto Rican version of the Psychosocial Impact of Assistive Device Scale (PR-PIADS) [25] with Hispanic older adults.

## 2. Materials and Methods

Approval from the Institutional Review Board (IRB) of the Medical Sciences Campus, University of Puerto Rico, was obtained prior to the beginning of this research. We used a cross-sectional descriptive study design [26] to identify AT that the sample already use, would not use, and would use if they had the device.

### 2.1. Participants’ Recruitment and Sampling

We recruited a non-probability convenience sample of 60 individuals from rural as well as urban areas in Puerto Rico. Inclusion criteria were (a) Hispanic men and women 70 years and older; (b) living independently in the community; (c) not receiving home care; (d) reported the need for help or difficulties with two instrumental activities of daily living (IADL) or one or more activities of daily living (ADL); and (e) no severe cognitive impairment as evidenced by a score of 24 or greater in the Minimental State Examination (MMSE) as recommended by the literature [27]. Exclusion criteria were (a) non-Hispanics; (b) institutionalized older adults; (c) individuals with dementia and severe impairments that require specialized AT equipment. Specialized AT equipment was defined as AT devices used to compensate for severe physical, communication, or sensory disabilities such as mobile hoist lifters, augmentative and alternative communication devices, and computer screen readers.

We posted flyers in locations frequently visited by older adults, such as senior centers, churches, and doctors’ offices. We also used a snowball sampling procedure. Interested participants were asked to call the researchers of this study to determine their eligibility, and if appropriate for the study, agree an appointment for the administration of the study’s assessment tools at a private location of their choice (i.e., at their home).

### 2.2. Data Collection Instruments

#### 2.2.1. The Socio-Demographic Questionnaire

A questionnaire was designed by the researchers of this study to describe the participants of this study. It gathered information about age, sex, education level, medical condition, place of living, and monthly income.

#### 2.2.2. Assistive Technology Card Assessment (ATCA)

The ATCA was developed for the purpose of this study using a methodological research design to test its content validity with aging experts and community-living older adults. The first step consisted of a systematic review conducted in PubMed, Medline, EbscoHost, PsycInfo, CINAHL, and AgeLine databases of the existing literature from 1999 to 2013 to identify AT devices used by community-living older adults that could be included in the questionnaire. A list of 110 relevant ATs from the literature was generated and organized into 16 categories. After an analysis of the frequency that each device was included on the assessed literature, the researchers generated a second list of 49 assistive technology devices (being the most used as stated by the literature). These devices were included in the first draft of the questionnaire and divided into 11 categories of AT. This draft of the questionnaire items, the AT devices glossary, and the set of instructions that was generated by the research team were sent to a group of five experts in aging and AT for content validity testing using the content validity ratio (CVR) exercise to determine the AT devices that would be included in the final version. The instrument was then tested by a sample of 10 community-living older adults. A debriefing interview with open-ended questions was administered to the participants to explore their opinions related to the instrument clarity, understandability, structure, and its utility to assess the AT needs of older adults. Following the recommendations made by the panel of experts and the sample of older adults, the researchers decided to eliminate four AT devices (adapted cutting board, audio books, hand-held magnifying glass with light, and text-to-speech program) and add five AT devices (security rug tape, night light with dark sensor, bed raisers, toilet base risor, and wheeled cart). Additional changes were made to the glossary including clarifying the description of some of the AT devices, substituting some of the images to increase their clarity, and increasing the font size of the ATCA text. The final ATCA included 50 cards depicting pictures of older adults using AT devices in 11 categories: AT for reading, AT for Mobility, AT for Personal Hygiene, AT for Toilet use, AT for Cooking, AT for Home Care, AT for Medication Management, AT for Communication, AT for Home Accessibility, and AT for Home Security. The participants sort these cards into the following labels: “I use this device, I have this device but do not use it” “I would not use this device” and “I would use this device but do not have it” For the purpose of this study, we selected the AT devices sorted in the category of “I would use this device, but do not have it” to describe the AT needs of the sample of this study. The researchers were available to help participants complete the ATCA. A glossary featuring each AT with a definition was provided for each participant to assist him or her understand the use of the device depicted in the cards.

#### 2.2.3. Puerto Rican Version of the Psychosocial Impact of Assistive Devices Scale (PIADS)

The original Psychosocial Impact of Assistive Devices Scale (PIADS) was designed with the purpose of addressing the need to measure the psychosocial impact of the person with the use of technological assistance [13]. It consists of a scale with 26 items, using a scoring system from −3 “maximum negative impact” to +3 “maximum positive impact” to indicate the extent to which the AT user is affected by using his or her device. Specific instrument sub-scales include competence (12 items), adaptability (six items) and self-esteem (eight items). PIADS has proven to be a reliable, valid and responsive measure with good clinical utility [28]. It is a responsive measure and sensitive to important variables such as the user's clinical condition, device stigma, and functional features of the device. The scale seems to have the power to predict the abandonment and retention of an assistive device [13]. The PR-PIADS was developed using standard procedures to culturally adapt the Spanish Spain version of the PIADS for the Puerto Rican population, demonstrating evidence of content validity [25]. The PR-PIADS has demonstrated evidence of content validity in the areas of semantic, content, idiomatic, and technical equivalence with the original version [25].

### 2.3. Procedures

During the first telephone contact with the participant, the researchers assessed the first four inclusion criteria. The researchers coordinated an individual face-to-face meeting with those who met these first four criteria. During this meeting, the researchers provided the participants with a full explanation of the study and the consent form. After addressing participant’s questions and obtaining signed consent the researchers administered the MMSE. Those participants who obtained the cut off score of 24 or above on the MMSE were included in this study. All of this participants were then asked to complete a socio-demographic questionnaire followed by the administration of the Assistive Technology Card Assessment, where participants were asked to sort each card into a single category depending on their perspective about it. Finally, the researchers administered to the participants the PR-PIADS using an interview format instead of a self-report measure as recommended by the PIADS manual. This decision was taken by the researchers of this study based on the cultural preference of this population to engage in a personal relationship provided by the interview format.

### 2.4. Data Analysis

Data from the socio-demographic questionnaire, the ATCA, and the PR-PIADS was analyzed using univariate analysis of central tendency descriptive statistics: mean and standard deviations for the continuous variables and frequency and percentages for categorical variables.

## 3. Results

### 3.1. Sample Characteristics

Sixty participants from different community sites aged 70–97 years met the inclusion criteria. The participants were predominantly female, had an educational level of high school or less, and had a low income of $1000 or less. The predominant health conditions reported by the participants were hypertension, musculoskeletal problems, and diabetes. See Table 1 for further results.

### 3.2. Assistive Technology Needs

The top five AT devices most frequently identified by participants as “I would use this but do not have it” were: non-skid jar openers, seat lifts, laundry basket with wheels, nonslip rubber mat, and “shopping cart with wheels”. Refer to Table 2 for further results.

The top three most frequently identified categories of AT from “I would use this but do not have it” were devices for cooking, devices for home tasks, and devices for home safety. Refer to Table 3 for the results of all the categories of AT devices and Table 4 for the specific devices included in each device category.

### 3.3. Psychosocial Impact of AT

The psychosocial impact of assistive devices used by the sample was as perceived as positive, deeming from their ratings (Table 5). The mean scores for the PIADS sub-scores were positive. The self-esteem sub-score showed lower ratings than all the other sub-scores.

## 4. Discussion

In this study the authors sought to describe the AT needs and psychosocial impact of AT of a sample of older adults living in Puerto Rico and to identify methodological challenges and lessons learned in using the Puerto Rican version of the PR-PIADS with this population. The participants of this study provided evidence that older adults face unmet needs for AT devices that could support their performance and participation in daily living activities. The results of this study also demonstrated that AT appears to have a positive impact on the perceived quality of life of community-living Hispanic older adults with functional limitations.

### 4.1. Assistive Technology Needs

The participants identified needs for AT devices predominantly in the categories of cooking, home tasks, and home safety. Specifically, the top three devices that the participants reported that they would use it if they have them were jar openers, seat lifts, and laundry baskets with wheels. These devices compensate for diminished energy and strength in the performance of daily activities. It is well known that the physiological effects of aging, such as loss of strength and endurance, might decrease tolerance for performing physically demanding activities [29,30]. The findings of this study are consistent with the findings of Cheek Nikpour and Heather [29] in which older adults from Brazil demonstrated a significant unmet need for assistive devices to compensate for energy and strength deficits in the performance of basic and instrumental activities of daily living. However, the results of the current study differ from the results of the study conducted by Gitlow and her colleagues [31] in exploring the AT needs of 57 community-dwelling older adults from Tompkins County. Gitlow stated that the most frequently identified needs existed in the categories of aids for hearing, aids for laundry, and aids for vision. These findings highlight the variation that exists among the needs of different older populations, thus requiring a client-centered approach when assessing AT needs.

Moreover, our study findings related to the socio-demographic characteristics of the sample are consistent with previous study results examining the disparities in usage of AT among people with disabilities. As such, the participants of this study were predominantly older women, with low educational levels and low monthly income. An early study showed that female gender was associated with a decreased likelihood of using AT devices, suggesting having a higher need for access to AT devices [15]. Previous studies have also found that having lower educational levels, lower household income, and later disability onset significantly put people at disadvantage in accessing and using AT devices [16,32]. These findings highlight the need for approaches to expand the usage of AT as well as to promote equal access to AT devices that enable greater autonomy and social participation for older people from disadvantaged populations.

### 4.2. Psychosocial Impact of AT

Our findings showed a positive psychosocial impact for assistive devices used for daily living activities. This finding comes from a sample that predominantly reported having hypertension and musculoskeletal disorders. This validates the assumption that assistive technologies help older people with mobility disabilities increase their quality of life and adapt or cope better with age-related functional disabilities. The self-esteem sub-score, although positive, showed lower ratings than all the other sub-scores. This result is consistent with previous studies conducted with individuals with neuromuscular disorders [33] and multiple sclerosis [34,35] using mobility devices, those with amyotrophic lateral sclerosis using wheelchairs, communication devices and environmental control units [36], and those with stroke [19] using a variety of AT devices. Social stigma associated with AT use has extensively been reported in the literature as a barrier to the uptake of AT devices [7,37–39]. It has been argued that the willingness to use assistive devices will depend on whether it supports or undermines the personal identity and self-image of the individual [7]. In the qualitative study conducted by Resnik and his colleagues [40], users of mobility devices expressed feelings of shame for needing help and felt that people with mobility problems were not seen as normal. Similarly, in a systematic review about the barriers older adults find for using AT devices, it was found that the participants were worried that people may perceive them to be in poor health or frail if they use AT devices [41].

### 4.3. Methodological Challenges and Lessons Learned

There were some culturally-based methodological concerns that emerged during the administration of the PR-PIADS to Hispanic older adults who live in Puerto Rico related to the format of administration, level of abstraction, and structure of the questionnaire. Most of the participants were reluctant to use a self-administered format (as recommended in the PIADS manual) to fill the PR-PIADS. These participants preferred a personal relationship provided by the interview format. The sample preference for an interview format instead of self-administration could be explained by two main factors. First, literacy issues could have played a role since 80% of the participants reported educational levels of high school or less. Second, the Hispanic population preference of a personal relationship or “personalismo” could have also been an influential factor. Since Hispanics expect health providers to be warm, friendly, and personal as well as to take an active interest in the patient's life [42], an interview format constitutes a perfect fit in the administration of the PR-PIADS for older Hispanics.

As to the level of comprehension, some of the participants also demonstrated poor understanding of the graded numerical response format indicated in the manual of the PIADS original version from −3 to +3. The response trends of these participants were to use positively skewed extreme responses (excessive use of positive endpoints of the PR-PIADS). The results support earlier studies that indicate the use of Likert scales among immigrant Latinos is often problematic [43] and that Hispanic Americans are more likely to agree with a given item, preferring extreme responses on rating scales more than non-Hispanic Americans do [44]. Hence, instructions and scale items may need to be evaluated for their ability to be understood by older Hispanic respondents.

Similarly, most of the participants demonstrated difficulty with the level of abstraction required by the PR-PIADS instructions. The PIADS original version’s instructions require making distinctions between the extents of the impact each device has made in the participant’s life in each of the PR-PIADS items. For example, when asked the abstract question about “How your assistive devices make you feel in relation to independence?” with numerical response options, rather than appear indecisive, they tended to give “very” or “somewhat” answers. Preferred were straightforward questions and categorical response options, for example, “Does your assistive technology make you feel more independent?” with responses options from very much to not at all. Difficulty in this sample’s level of comprehension could be explained by the low educational level of the majority of the participants of this study. Based on these findings, we suggest changing the type and the direction of the numerical Likert scale to categorical responses when used with older Hispanics with low educational levels. We also suggest to first ask participants to answer "Yes" or "No" to the question of how much have their devices make them feel in relation to each PIADS item. For example, the interview question related to the PIADS item of “competence” will be: “Has your AT device made you feel more competent?” If the answer is "Yes", the participant is then instructed to indicate (on the scale from “very much” to “not at all”) how the AT device makes them feel in relation to the PIADS item of competence.

Finally, some of the participants also demonstrated difficulty in understanding how to answer the PR-PIADS items when they were asked to use the structure of the list format. The instructions of the PR-PIADS ask the participants to describe how an assistive device affects their life and makes them feel in relation to each of the PR-PIADS items. Participants constantly asked for repetition of the instructions for each of the items. Therefore, it is recommended that a modified script of the instructions to answer the PR-PIADS be provided and repeated in each of the PR-PIADS items to increase understandability and recording of the instructions. For example, instead of item number five being “confusion”, it can be enhanced to “Does your assistive technology makes you feel more confused?”

In spite of these methodological challenges, this study’s results indicated that the PR-PIADS is still useful for assessing quality of life effects attributable to AT among Hispanic older adults living in Puerto Rico. It also provided valuable information that can be used to evaluate the effectiveness of AT to enhance its users’ competence, adaptability and self-esteem.

### 4.4. Limitations of the Study

The results may have limited generalizability to other populations and locations due to the use of a convenience snowball sample and small sample size. Further, the ATCA was not tested for construct validity and reliability. Therefore, if this questionnaire is used again, different results could be obtained. Moreover, the ATCA did not include all the range of AT devices currently available for the older population. Therefore, this study was not able to identify the sample’s needs for other AT devices that were not included in the ATCA questionnaire.

## 5. Conclusions

Older Hispanics living in Puerto Rico (PR) have unmet needs for using AT devices to overcome difficulties in daily living activities. Users perceived that the AT device enabled them in positive ways, in particular use made them feel more competent and that they have the ability to cope with functional disabilities in daily life activities. As AT devices are a fundamental environmental factor to maintain independence in different activities, it is important to apply tools such as the PIADS in clinical practice. However, based on our findings, adaptations to the PR-PIADS should be conducted to overcome the culturally-related instrumentation challenges found in this study and to increase the cultural validity of the obtained data for older Hispanics.

Future studies should determine the association between the socio-demographic characteristics of older Hispanics with the need for using AT devices. Further studies are also needed to investigate why people with low socio-economic levels are not obtaining the AT that they identified that they need in this study. Investigating why older Hispanics do not have the devices that they need may helps inform the need for policy change. Moreover, a follow-up methodological study should be conducted to culturally adapt the PR-PIADS with older populations and provide evidence of its psychometric properties.

## Figures and Tables

**Figure:**
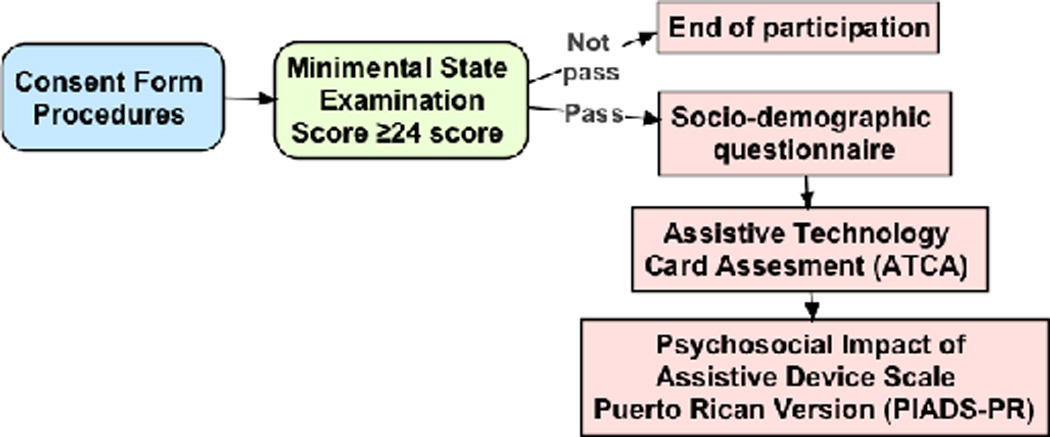


**Table 1:** Socio-demographic characteristics of the participants.

Socio-Demographic Characteristics	Total *n* = 60
Age Range (Min, max)	70–97
Mean (SD)	77 (6.27)
Sex, *n* (%)	
Female	40 (66.7)
Male	20 (33.3)
Educational Level, *n* (%)	
High school or less	48 (80)
Some college education	12 (20)
Monthly Income, *n* (%)	
Low(<$1000)	50 (83)
Medium ($1000–$2000)	8 (13)
High (>$2000)	2 (3)
Health Conditions, *n* (%)	
Hypertension	35 (58)
Musculoskeletal	33 (55)
Diabetes	32 (53)
Visual	15 (25)
Respiratory	13 (23)
Cardiac	12 (20)
Overweight	12 (20)

Min = Minimun; Max = Maximum; SD = Standard Deviation

**Table 2:** Assistive technology devices identified as “I would use this but do not have it”.

Assistive Technology	Number and Percentages of Responses That Reported “I Would Use This but Do Not Have It” *n* (%)
Jar Openers	35.0 (58.3)
Seat Lift	34.0 (56.7)
Laundry Basket with Wheels	30.0 (50.0)
Nonslip Rubber Mat	29.0 (48.3)
Shopping Cart on Wheels	29.0 (48.3)
Reacher	28.0 (46.7)
Adhesive Tape to Stabilize Rugs	27.0 (45.0)
Long- Handle Cleaning Brush	26.0 (43.3)
Emergency Alert System	26.0 (43.3)
Medications Reminder	25.0 (41.7)
Text Enlarger	24.0 (40.0)
Magnifier that I do Not Have to Hold	22.0 (36.7)
Bed Or Chair Lifts	22.0 (36.7)
Long-Handle Shoe Horn	22.0 (36.7)
Sock Aid	22.0 (36.7)
Long-Handle Sponge	21.0 (35.5)
Electric Can Opener	21.0 (35.5)
Rails Around Toilet	19.0 (31.7)
Locator Keys	18.0 (30.0)
Nail Clipper with Magnifier	17.0 (28.3)
Nonslip Mat	17.0 (28.3)
Dressing Stick	17.0 (28.3)
Long-Handle Dustpan	17.0 (28.3)
Remote Controls for Electrical Equipment	16.0 (26.7)
Tub Bench	15.0 (25.0)
Button Hook	15.0 (25.0)
Handle for Carry Bags	15.0 (25.0)
Hand Shower	13.0 (21.7)
Raised Toilet Base	13.0 (21.7)
Text Enlarger for PC, Tablet or Cellular	11.0 (18.3)
Rail for Bed	11.0 (18.3)
Simple Cellular	11.0 (18.3)
Simple TV Remote Control with Large Buttons	11.0 (18.3)
High Stool with Long Handle	11.0 (18.3)
Long-Handle Duster	10.0 (16.7)
Scooter	9.0 (15.0)
Three-in-one Commode	9.0 (15.0)
Lever Knobs	9.0 (15.0)
Walker	8.0 (13.3)
Amplified Phone	8.0 (13.3)
Grab Bars	7.0 (11.7)
Raised Toilet Seat	6.0 (10.0)
Hand Held Magnifier	5.0 (8.3)
Night Light	5.0 (8.3)
Wheelchair	3.0 (5.5)
Phone with Amplified Keys	3.0 (5.5)
Glasses	2.0 (3.3)
Cane	1.0 (1.7)
Pill Organizers	1.0 (1.7)

**Table 3:** Most frequently identified categories of assistive technology devices identified as “I would use this but do not have it”.

Categories	Percentage of Total Responses (%) Categories on “ I Would Use This but Do Not Have It”
Home Tasks	37.3
Home Safety	32.2
Dressing	31.7
Home Accessibility	25.7
Personal Hygiene	25.0
Medication	21.7
Reading	21.3
Mobility	20.9
Toileting	19.6
Communication	12.2

**Table 4:** Most frequently identified devices in the top three categories of assistive technology identified as “I would use this but do not have it”.

Category	Assistive Technology Reported “I Would Use This but Don’t Have It”	Number and Percentages of Responses that Reported “I Would Use This but Don’t Have It” *n* (%)
Cooking	Jar Openers	35 (58.3)
	Nonslip Rubber	29 (48.3)
	Built-up Handles for Utensils	27 (45.0)
Home Tasks	Laundry Basket on Wheels	30 (50.0)
	Shopping Cart on Wheels	29 (48.3)
	Long-handle Cleaning Brush	26 (43.3)
Home Safety	Adhesive tape to stabilize rug	27 (45.0)
	Emergency Alert System	26 (43.3)
	Night Light	5 (8.3)

**Table 5:** Mean and standard deviations scores in each sub-scale of the Puerto Rican Psychosocial Impact of Assistive Device Scale.

PR-PIADS Scale (*n* = 60)	Study Mean	SD
Competence	2.77	0.45
Adaptability	2.51	0.61
Self-esteem	1.98	0.49
